# Diagnosis of knee meniscal injuries using artificial intelligence: A systematic review and meta-analysis of diagnostic performance

**DOI:** 10.1371/journal.pone.0326339

**Published:** 2025-06-24

**Authors:** Soheil Mohammadi, Ali Jahanshahi, Mohammad Shahrabi Farahani, Mohammad Amin Salehi, Negin Frounchi, Ali Guermazi

**Affiliations:** 1 Mallinckrodt Institute of Radiology, Washington University in St. Louis, Saint Louis, Missouri, United States of America; 2 Social Determinants of Health Research Center, Trauma Institute, Guilan University of Medical Sciences, Rasht, Iran; 3 Faculty of Medicine, Guilan University of Medical Sciences, Rasht, Iran; 4 Medical Students Research Committee, Shahed University, Tehran, Iran; 5 Research Institute, McGill University Health Centre, Montreal, Quebec, Canada; 6 Kidney Research Center, Tabriz University of Medical Sciences, Tabriz, Iran; 7 Department of Radiology, VA Boston Healthcare System, Boston University School of Medicine, Boston, Massachusetts, United States of America; Assiut University Faculty of Medicine, EGYPT

## Abstract

**Aim of the study:**

The aim was to systematically review the literature and perform a meta-analysis to estimate the performance of artificial intelligence (AI) algorithms in detecting meniscal injuries.

**Materials and methods:**

A systematic search was performed in the Scopus, PubMed, EBSCO, Cinahl, Web of Science, IEEE Xplore, and Cochrane Central databases on July, 2024. The included studies’ reporting quality and risk of bias were evaluated using the Transparent Reporting of a multivariable prediction model for Individual Prognosis or Diagnosis (TRIPOD) and the Prediction Model Study Risk of Bias Assessment Tool (PROBAST), respectively. Also, a meta-analysis was done using contingency tables to estimate diagnostic performance metrics (sensitivity and specificity), and a meta-regression analysis was performed to investigate the effect of the following variables on the main outcome: imaging view, data augmentation and transfer learning usage, and presence of meniscal tear in the injury, with a corresponding 95% confidence interval (CI) and a P-value of 0.05 as a threshold for significance.

**Results:**

Among 28 included studies, 92 contingency tables were extracted from 15 studies. The reference standard of the studies were mostly expert radiologists, orthopedics, or surgical reports. The pooled sensitivity and specificity for AI algorithms on internal validation were 81% (95% CI: 78, 85), and 78% (95% CI: 72, 83), and for clinicians on internal validation were 85% (95% CI: 76, 91), and 88% (95% CI: 83, 92), respectively. The pooled sensitivity and specificity for studies validating algorithms with an external test set were 82% (95% CI: 74, 88), and 88% (95% CI: 84, 91), respectively.

**Conclusion:**

The results of this study imply the lower diagnostic performance of AI-based algorithms in knee meniscal injuries compared with clinicians.

## Introduction

The menisci are crescent-shaped fibrocartilaginous structures in the knee joint, vital for weight-bearing, shock absorption, and leg movements [1,2]. Meniscal damage is considered the most frequent knee injury and can be either traumatic or degenerative. Meniscal tears and macerations are the two types of meniscal damage that can lead to pain, functional limitations, and early onset progression of osteoarthritis. Therefore, rapid and accurate diagnosis of meniscal damage is of paramount importance as it enables early detection and prevention of osteoarthritis [3,4].

Magnetic resonance imaging (MRI) is considered the noninvasive modality of choice for the diagnosis of meniscal damage, while arthroscopy is highly accurate but an invasive method [5]. Increased signal intensity in MR images alongside soft tissue swelling can be interpreted as meniscal tear, although depending on the type of tear, various signs might be present [6]. A recent meta-analysis on the diagnostic performance of MRI in meniscal injuries found that time for acquisition, MRI system technology, and type of MRI sequence can affect the performance of this modality [7]. Additionally, previous studies have shown that the accuracy of MRI is markedly poorer in specific types of meniscal damage, such as degenerative tears and macerations, and some cases of meniscal tears accompanying anterior cruciate ligament tears [8]. Furthermore, due to the time-consuming acquisition and interpretation process of MRI, as well as inter-reader and intra-reader variability, its diagnostic performance can vary widely [9]. These factors highlight the potential artificial intelligence-based methods for the interpretation of images to improve early detection of meniscal damage.

Artificial intelligence (AI) is one of the vast branches of computer science that enables the accomplishment of various tasks without human intervention [10]. Machine learning is a component of AI in which algorithms are designed and programmed to learn tasks from data through experience [11]. Deep learning, which is structured similarly to neuronal networks, is a subset of machine learning that processes data through multiple layers so that the output of one layer serves as the input for the next layer [11,12].

The utilization of AI in medicine is rapidly growing today [13]. Previous studies have demonstrated that deep learning algorithms can perform thoroughly with acceptable accuracy, especially in diagnostic tasks [12]. A study by Xue et al. [14] observed a higher sensitivity, specificity, and AUC in deep learning-aided image-based cancer diagnosis (88%, 88%, and 0.94, respectively). Also, Kuo and colleagues [15], in a study comparing clinicians and AI in fracture detection, showed that AI performance is the same as clinicians’.

In various medical situations, such as meniscal damage, timely and accurate detection can significantly enhance the outcome. AI-assisted diagnosis has the potential to provide substantial benefits in terms of both time and cost. In this study, the aim was to systematically review the literature and perform a meta-analysis to estimate the performance of artificial intelligence algorithms in detecting meniscal injuries.

## Materials and methods

### Protocol and registration

This study was performed based on Preferred Reporting Items for Systematic Reviews and Meta-Analyses (PRISMA) guidelines with a major focus on Diagnostic Test Accuracy extension (PRISMA-DTA) and adherence to the preferred essential items in writing a systematic review of diagnostic test accuracy studies [16,17]. Our study was registered on the International Prospective Register of Systematic Reviews (PROSPERO) website (Registration No. CRD42022323106). The aim was to include studies assessing the performance of AI in detecting meniscal injuries. Two reviewers (Medical doctors with at least 4 years of experience in performing systematic reviews) independently worked on each step of this review, and any discrepancies were solved through the supervision of the third reviewer (A medical doctor with at least 6 years of experience in performing systematic reviews) in the discussion.

### Search strategy and study selection

Primarily, a comprehensive literature search was performed in databases of Scopus, PubMed, EBSCO, Cinahl, Web of Science, IEEE Xplore, and Cochrane Central in April, 2022 using related keywords (S1 Table), and our systematic search was updated in July, 2024 to find published articles that evaluated validation and performance of an AI algorithm as a diagnostic tool for detection of meniscal damage, regardless of their study setting, language, target population, and publication time. Also, all references of the included studies were screened to find any studies that might have been missed. The articles violating the following exclusion criteria were excluded from our study: letters, opinions, book chapters, conference abstracts, reviews, animal studies, studies performing only segmentation analysis, and studies using natural language processing (NLP) on electronic health records (EHR).

### Data extraction

The following data were extracted from the included studies: First author’s name and publication year, country, study design, their inclusion and exclusion criteria for the images or the participants, imaging modality, view of imaging, algorithms and architectures, dimension of images, evaluation and validation methods, validation size, number of images in each training, testing, and tuning sets, model output, the standard reference for diagnosis, values of true positive, true negative, false positive, false negative, sensitivity, specificity, positive and negative predictive values, the AUC, and accuracy. Moreover, an email was sent to the correspondence of the studies that had not reported their data thoroughly to ask for the complete results. The reported data were used to build contingency tables and calculate sensitivity and specificity, where applicable.

### Statistical analysis

In order to determine the diagnostic performance of AI and clinicians, a random-effect meta-analysis was performed with a corresponding 95% CI, using contingency tables extracted from the included studies for at least three adequate studies to calculate pooled sensitivity and specificity. A meta-regression analysis was conducted to determine between-study heterogeneity for the following covariates: view of imaging, usage of data augmentation and transfer learning, and presence of meniscal tear in the injury. Besides, the data were divided based on whether the injury was on the medial or lateral side and a separate meta-regression analysis was performed for each medial and lateral menisci. A P-value of 0.05 was considered as a threshold for significance. The entire data analysis was conducted using Stata 16 software (Stata Corp, College Station, TX) [18,19]. The “midas” command in Stata does not use hierarchical or bivariate models to simultaneously model sensitivity and specificity in the meta-analysis of diagnostic performance studies. Instead, it uses a univariate approach where the logit-transformed sensitivity and specificity are separately analyzed and combined across studies using a fixed-effects or random-effects model. It also uses a logistic regression model for the random-effects model and meta-regression in order to make comparisons between subgroups. This method has been widely used in previous meta-analyses [15,20].

### Publication bias

The slope coefficient represents the relationship between the diagnostic odds ratio (DOR) and the inverse of the square root of the effective sample size (ESS). Publication bias refers to the tendency for studies with positive or significant results to be more likely to be published than those with negative or nonsignificant results. In the context of diagnostic performance studies, publication bias can occur if studies reporting higher DORs are more likely to be published, leading to an overestimation of the test’s accuracy. The slope coefficient is used to assess the presence of publication bias by examining whether there is a relationship between the DOR and the ESS. If there is no publication bias, the slope coefficient should be close to zero. A positive slope coefficient suggests that studies with higher DORs have smaller ESS, indicating a potential publication bias towards studies with positive results. Conversely, a negative slope coefficient suggests a potential publication bias towards studies with negative results [21].

### Quality and risk of bias assessment

Since there are no perfectly suitable criteria to assess AI studies’ reporting quality and risk of bias, previous studies [15] were used to find the best tools for this attempt. The reporting quality of the included studies in reporting the results was assessed using the TRIPOD. This checklist consists of 22 items evaluating each section [22]. A modified version of TRIPOD was used due to the inapplicability of its few items for AI studies (S2 Table).

The risk of bias and applicability of the included studies were estimated using the PROBAST, consisting of several categorized questions in 4 domains of participants, predictors, outcomes, and analysis [23]. The predictors’ domain was omitted because it was irrelevant in the setting of our study, and both training and testing sets were assessed in the first domain (S3 Table).

## Results

### Study selection and characteristics

**Fig 1:**
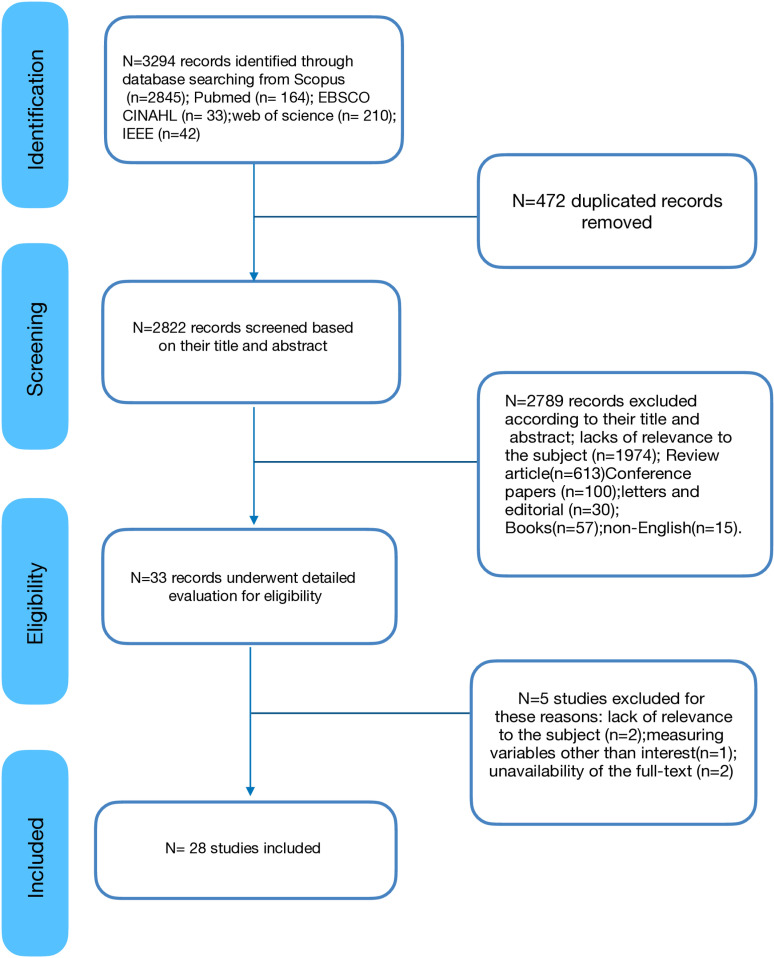
Flowchart of study selection.

As a result of our systematic search, 3294 studies were extracted and downloaded to Endnote version 20. After removing duplicates, 2822 studies underwent the title and abstract screening process. A full-text screening was carried out for 33 eligible studies, leading to the exclusion of five studies for these reasons: lack of relevance [24–26], unavailability of the full text [27], and measuring variables other than interest. At last, 28 studies [2,9, 28–53] included meeting the inclusion criteria. More detailed information on study selection could be obtained from the flow diagram in Fig 1.

All included studies used MRI to investigate meniscal damage, although one of them [36] also included computed tomography (CT) images in their study. Twenty-four studies [2,9,28–40,45–53] recruited an internal test set to estimate the diagnostic performance of their AI algorithm, of which two studies [2,38] had an external test set too. Four studies [41–44] used only an external test set for this purpose.

All of the MRI, except in two studies [42,49], were in sagittal view. Also, thirteen studies [2,9,28,31,33,36,39,40,44,45,51–53] included multiple views of MRI, and five studies [39,40,46–48] developed different models for each view of MRI. Regarding the type of injury, [28,30,32,33,35,40,42,45,51,53]eighteen studies (Four externally validating, and 14 internally validating studies) [2,9,29,31,34,36–39,41,43,44,46–50,52] did not report the subtype of the meniscal damage. Three studies (two with internal and one with both internal and external test sets) [28,40,45] reported degenerative meniscal injury. Two studies included complex tears [28,42], and three observed either partial or complete maceration of the menisci [30,35,42]. Furthermore, among the internal validation studies, two [30,32] had both vertical and horizontal tears in their dataset, while two studies [33,53] only noted horizontal tears.

Only five studies [39,40,44,49,53] used a cross-validation method to validate their AI algorithm, while others used different validation methods, namely random split sampling, stratified split sampling, validation set, or training set. The model output of 21 studies [2,29–35,37,41–44,46–53] was binary classification, of which one study [30] also had its model perform a multiclass classification on MRI. Among others, the study by Chou et al. [39] assessed the algorithm’s performance in determining the probability of meniscal damage. Eleven studies [2,28,31,33,35,39,42,44,45,50,53] had a comparison group of expert clinicians, thirteen [9,30,32,36–38,42,46–49,52,53] carried out a comparison of multiple algorithms, while one study [51] made a comparison between multiple imaging views. All of the included studies mentioned a reference standard for their diagnosis, of which three [32,41,43] were their past study, while others had experts such as radiologists or surgeons as the reference standard, although one study [46] did not state their reference standard. More detailed information can be obtained from Tables 1,2, and S4.

**Table 1:** Study characteristics, internal validation.

First author	Year	Country	Imaging modality	Target condition	Type of tear	MRI sequence planes	Comparison group	No. of Images per training Set	No. of Images per tuning Set	No. of Images per testing Set	Validation size	Model output	Reference standard
Bharath Ramakrishna	2008	China	MRI	Meniscus	Simple and complex meniscal tears, meniscal fragment, degenerative tear	Sagittal, Coronal	Expert clinicians	10	NR	160	NR	NR	Expert consensus
Mohammad Hossein Fazel Zarandi	2016	Iran	MRI	Meniscus	NR	Sagittal	NR	198	NR	50	NR	0 and 1 mode, Binary Classification	Expert consensus
Ahmet Saygılı	2018	Turkey	MRI	Meniscus	Horizontal tears, Vertical tears, Partial Maceration	Sagittal	Comparison of multiple algorithms	66	NR	22	NR	Binary Classification, Multiclass Classification	Expert consensus
Nicholas Bien	2018	USA	MRI	Meniscus	NR	Sagittal, coronal, axial	Expert clinicians	1130	120	NR	120	Binary Classification	Musculoskeletal radiologists
Vincent Couteaux	2019	France	MRI	Meniscus	Vertical tear, horizontal tear	Sagittal	Comparison of multiple algorithms	1128	NR	700	NR	Binary Classification	Past study
Benjamin Fritz	2020	Switzerland	MRI	Medial and lateral meniscus	Horizontal tears	Sagittal, coronal	Expert clinicians	18520	NR	1000	1000	Binary Classification	Expert consensus
Emre ÖLMEZ	2020	Turkey	MRI	Meniscus	NR	Sagittal	Comparison of multiple algorithms	NR, 100	NR	10, 50	NR	Binary Classification	Expert consensus
Alexander Tack	2021	Germany	MRI	Meniscus	NR	Sagittal	NR	DESS: 1200, IW TSE: 1197	NR	840	359	Binary Classification	Expert consensus
Bruno Astuto	2021	USA	MRI	Meniscus	Nondisplaced or Displace Tear, Partial Resection, Complete Maceration	Sagittal	Expert clinicians	1005	NR	215	215	Binary Classification	Expert consensus
Xubin Qiu	2021	China	MRI, CT	Meniscus	NR	Sagittal, horizontal	Comparison of multiple algorithms	2460	NR	2460	NR	NR	Expert consensus
Ali Can Kara	2021	Turkey	MRI	Meniscus	NR	Coronal, sagittal, axial	Comparison of multiple algorithms	1130	NR	120	120	Binary Classification	NR
Hyunkwang Shin	2022	Republic of Korea	MRI	Medial and lateral meniscus	NR	Coronal, sagittal	Comparison of multiple algorithms	583, 431, 348	NR	250, 185, 149	NR	NR	Orthopedic knee specialists
Jie Li	2022	China	MRI	Meniscus	Degenerative	Supine, sagittal, coronal	Expert clinicians	9072	NR	3600 for internal test set, 1620 for external test set	3960	NR	Radiologists/ Arthroscopic surgery
Yuan‑Zhe Li	2022	China	MRI	Medial and lateral meniscus	NR	Sagittal	Comparison of multiple algorithms	382	NR	164	NR	NR	Knee joint operation report
Truong Nguyen Khanh Hung	2022	China	MRI	Meniscus	NR	Coronal, sagittal	Expert clinicians	234	NR	200	150, 120	Binary Classification	Expert consensus
Yi-Ting Chou	2022	Taiwan	MRI	Meniscus	NR	Coronal, sagittal	Expert clinicians	90, 84, 93, 109, 648	NR	20, 17, 23, 33, 163	NR	Probability of tearing	Expert consensus
Yi Wang	2022	Taiwan	MRI	Meniscus	Degenerative	Coronal, sagittal	NR	114.8	NR	NR	49.2	NR	Expert consensus
Shilpa Sharma	2022	India	MRI	Meniscus	NR	Coronal, sagittal, axial	Comparison of multiple algorithms	1130	NR	120	120	Binary Classification	Expert consensus
Yingkai Ma	2023	China	MRI	Anterior and posterior meniscus	Horizontal tears	Coronal, sagittal	Comparison of multiple algorithms, Expert clinicians	1396	NR	200	NR	Binary Classification	Expert consensus
Anita Thengade	2023	India	MRI	Meniscus	NR	Coronal, sagittal, axial	Comparison of multiple algorithms	1130	NR	NR	120	Binary Classification	Expert consensus
Massimiliano Mangone	2023	Italy	MRI	Medial meniscus	Broken, radial, longitudinal, or fracture lines present in at least three slices or morphologic deformity	Coronal, sagittal, axial	Comparison of multiple views	466	NR	98	NR	Binary Classification	Expert consensus
Fatma Harman	2023	Turkey	FastMRI	Meniscus	NR	NR	Comparison of multiple algorithms	973	NR	118	199	Binary Classification	Expert consensus
Erdal Güngör	2024	Turkey	MRI	Meniscus	NR	Coronal, sagittal, axial	Comparison of multiple algorithms	450	NR	92	100	Binary Classification	Expert consensus
Kexin Jiang	2024	China	MRI	Meniscus	NR	Sagittal	Expert clinicians	1229	NR	351	176	Binary Classification	Expert consensus

MRI: Magnetic Resonance Imaging; NR: Not Reported

**Table 2:** Study characteristics, external validation.

First author	Year	Country	Imaging modality	Target condition	Type of tear	MRI sequence planes	Comparison group	No. of Images per training Set	No. of Images per tuning Set	No. of Images per testing Set	Validation size	Model output	Reference standard
Cemal Kose	2007	Turkey	MRI	Meniscus	NR	Sagittal	NR	500	NR	100	NR	Binary Classification	Past study
Valentina Pedoia	2018	USA	MRI	Meniscus	Displaced or complex tears without deformity, maceration of the meniscus	NR	Comparison of multiple algorithms, expert clinicians	961	NR	296	221	Binary Classification	Expert consensus
Victoire Roblot	2019	France	MRI	Meniscus	NR	Sagittal	NR	1123	NR	700	NR	Binary Classification	Past study
Benoit Rizk	2021	Switzerland	MRI	Meniscus	NR	Coronal, sagittal	Expert clinicians	6221, 904	NR	299.120	1538,226	Binary Classification	Expert consensus
Jie Li	2022	China	MRI	Meniscus	Degenerative	Supine, sagittal, coronal	Expert clinicians	9072	NR	3600 for internal test set, 1620 for external test set	3960	NR	Radiologists/ Arthroscopic surgery
Truong Nguyen Khanh Hung	2022	China	MRI	Meniscus	NR	Coronal, sagittal	Expert clinicians	234	NR	200	150, 120	Binary Classification	Expert consensus

MRI: Magnetic Resonance Imaging; NR: Not Reported

### Study participants

The number of participants represented by the training data in each study ranged from 28 [29] to 7903 [44] (median, 530.50; interquartile range, 963; S4 Table). The percentage of disease-positive participants varied widely (median, 42.65; range, 87; interquartile range, 32.92; S4 Table). However, six studies [9,32,34,37,43,49] did not report data on the number of participants, and eleven studies [2,32,35,37,38,40,42,44–46,49] did not report the proportion of disease-positive participants.

### Algorithm development and model output

The number of images in training sets (median, 583; range, 18510; interquartile range, 1015.2; Table 1) and testing sets (median, 154.50; range, 3590; interquartile range, 182.50; Table 1) differed widely among the included studies, although one of the studies developing an internally validated algorithm [31], did not report the size of their testing set. The size of the datasets in the externally validating studies varied from 100 [41] to 1620 [45] (median, 296; interquartile range, 580; Table 2). Ten studies [34,35,39,42,43,45,46,48–50] used data augmentation, and five [29,31,37,46,47] carried out a transfer learning process.

The performance of the presented algorithms in the studies was assessed by various metrics such as accuracy (n = 19), sensitivity and specificity (n = 19), negative or positive predictive value (n = 7), AUC (n = 18), and f1 (n = 2). More detailed information on algorithm development is shown in Tables 1,2, and S4–S6.

### Quality assessment

**Fig 2:**
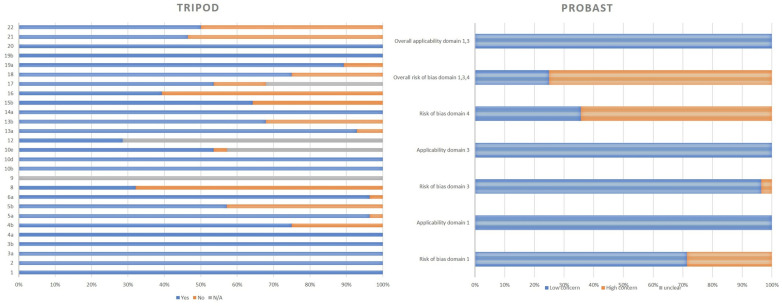
Adherence to Transparent Reporting of a Multivariable Prediction Model for Individual Prognosis or Diagnosis (TRIPOD), and Prediction Model Study Risk of Bias Assessment Tool (PROBAST) reporting guidelines.

In terms of adherence to the TRIPOD checklist, four applicable items were reported in less than or equal to 50% of the studies: sample size estimation, reporting the model’s performance, availability of supplementary information, and declaration of the funding source. Fig 2 demonstrates the pattern of articles’ adherence to this tool.

According to the PROBAST checklist, more than 70% of the studies were found to have low concern regarding the risk of bias and applicability in participant selection and outcome determination. However, there was a high concern in the analysis domain for more than 60% of the included studies. The overall results show that although all of the studies were at low risk in applicability, less than 30% of the studies were found to be at lower risk of bias based on this tool (Fig 2).

### Meta-analysis

Of the 28 included studies, 15 [2,9,28–31,33,35,38,39,41,42,44,45,53] provided sufficient data and 92 contingency tables were extracted from them. Seventy contingency tables were extracted from 12 [2,9,28–31,33,35,38,39,45,53] studies, internally validating the AI algorithm. Five tables were extracted from four [2,41,42,44] external validation studies and 17 tables from four studies [28,31,33,38] assessing the clinicians’ diagnostic performance on internal test sets. Furthermore, 24 contingency tables were extracted from five studies internally validating AI algorithms [9,28,30,33,39], and six tables from two studies evaluating clinicians’ performance on internal test sets [28,33] presenting data for lateral and medial menisci separately.

The pooled results are shown in Table 3. The pooled sensitivity and specificity for internally validated algorithms were 81% (95% CI: 78, 85), 78% (95% CI: 72, 83), and for clinicians, 85% (95% CI: 76, 91), and 88% (95% CI: 83, 92), respectively. On the other hand, the pooled sensitivity and specificity for studies validating algorithms with an external test set were 82% (95% CI: 74, 88) and 88% (95% CI: 84, 91), respectively. Besides, a subgroup analysis was performed on the studies reporting separate lateral and medial menisci results. Table 3 presents detailed pooled results of our meta-analysis.

**Table 3:** Pooled sensitivities, specificities, and area under the curve, positive likelihood ratio, negative likelihood ratio, diagnostic odds ratio.

Parameter	Sensitivity (%)	Specificity (%)	AUC	Positive Likelihood Ratio	Negative Likelihood Ratio	Diagnostic Odds Ratio	No. of Contingency Tables
**Algorithms Internal Validation, All studies**	81 (78–85)	78 (72–83)	0.86 (0.83–0.89)	3.7 (2.8–5)	0.23 (0.17–0.29)	16 (10–27)	70
Medial meniscus	83 (77–87)	82 (74–89)	0.88 (0.85–0.91)	4.7 (3.0–7.3)	0.21 (0.15–0.29)	22 (12–44)	24
Lateral Meniscus	65 (56–74)	63 (52–74)	0.67 (0.63–0.71)	1.8 (1.2–2.5)	0.55 (0.39–0.77)	3 (2–6)	24
**Algorithms External Validation, All studies**	82 (74–88)	88 (84–91)	0.9 (0.87–0.92)	6.9 (5–9.5)	0.20 (0.13–0.30)	34 (18–64)	5
**Clinicians Internal Validation, All studies**	85 (76–91)	88 (83–92)	0.93 (0.90–0.95)	7.1 (4.9–10.1)	0.17 (0.11–0.27)	41 (22–78)	17
Medial meniscus	95 (90–97)	88 (81–92)	0.97 (0.95–0.98)	7.8 (5.1–11.9)	0.06 (0.03–0.11)	133 (91–194)	6
Lateral Meniscus	70 (62–77)	90 (78–96)	0.72 (0.68 – 0.76)	6.9 (3.0–16.0)	0.34 (0.26–0.43)	21 (8–54)	6

AUC: Area under curve

**Fig 3:**
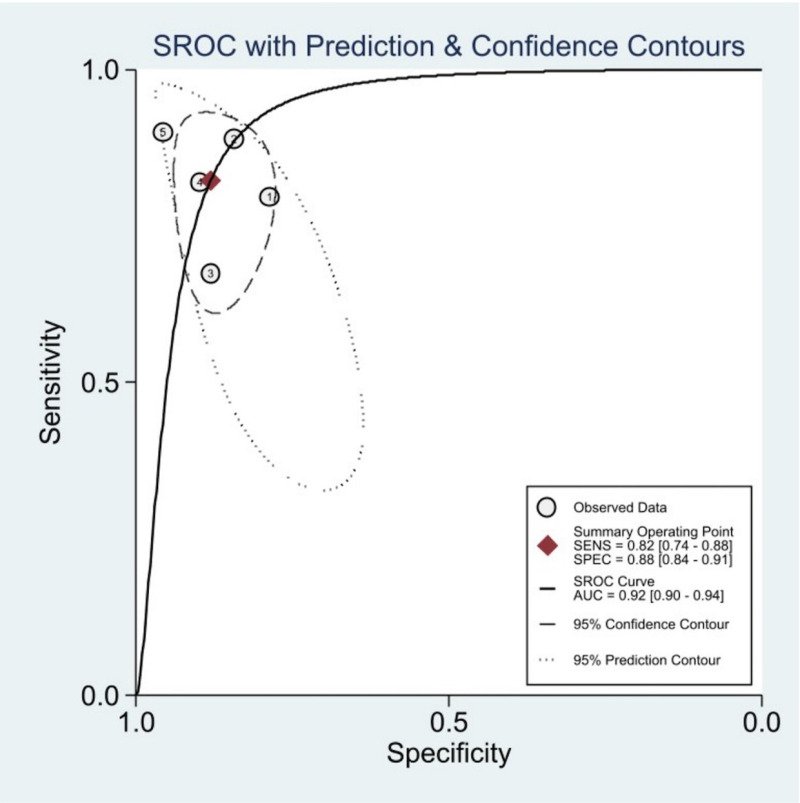
SROC curves.

**Fig 4:**
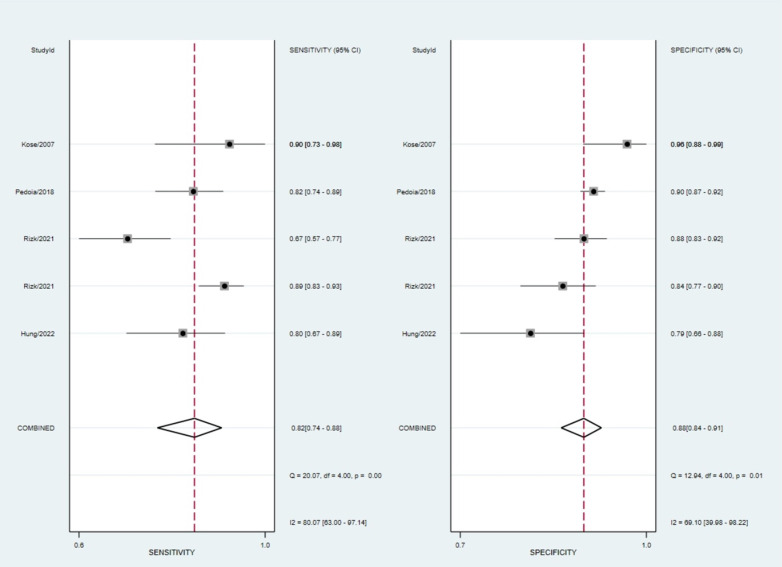
Forest plots.

Regarding meta-regression analysis, for studies internally validating their algorithm, statistically significant higher sensitivity was associated with data augmentation usage (85%; 95% CI: 77, 93; P, 0.01) and multiple-view imaging (86%; 95% CI: 81, 90; P, 0.00). In contrast, no significant difference was observed in the usage of transfer learning. Moreover, lower specificity was associated with the presence of tear in the injury (87%; 95% CI: 84, 90; P, 0.00) and multiple view imaging (85%; 95% CI: 81, 89; P, 0.00) in the studies validating algorithms with external test sets. There was no significant difference between multiple and single-view imaging in the studies assessing clinicians’ performance on internal test sets. S7–S11 Tables illustrate more detailed results, including the meta-regression analysis of studies reporting lateral and medial menisci separately. Also, SROC curves and Forest plots for each analysis are included in Figs 3 and 4, respectively.

### Publication bias

**Fig 5:**
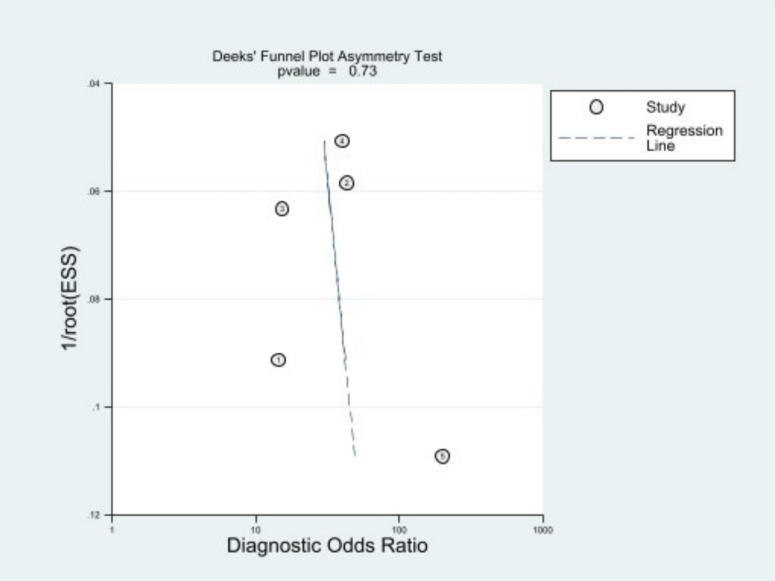
Deek’s funnel plots.

The result of the publication bias analysis is presented in S12 Table. The slope coefficient for studies evaluating internally and externally validated algorithms and clinicians’ performance on internal test sets were 0.12 (95% CI: −6.18, 6.44; P, 0.967), 8.39 (95% CI: −63.24, 80.04; P, 0.734), and −14.83 (95% CI: −24.81, −4.85; P, 0.006), respectively. Therefore, there is a high risk of publication bias for studies assessing clinicians’ performance with internal test sets. Regarding the publication bias results, funnel plots included in Fig 5 are more illustrative and informative.

## Discussion

The main findings of our study are as follows: AI algorithms had a lower diagnostic accuracy compared with clinicians on internal validation, with pooled sensitivity and specificity of 81% (95% CI: 78, 85) and 78% (95% CI: 72, 83), respectively. Almost the same pattern was applied to the medial and lateral menisci. Still, interestingly, AI algorithms reached a better diagnostic accuracy for medial meniscus damage than lateral meniscus, with pooled sensitivity and specificity of 83% (95% CI: 77, 87) and 82% (95% CI: 74, 89). Regarding external validation, the diagnostic accuracy of AI algorithms was not comparable with clinicians because no studies evaluated clinicians’ performance on external test sets. However, AI showed an acceptable pooled sensitivity and specificity of 82% (95% CI: 74, 88) and 88% (95% CI: 84, 91), respectively. Eleven internal validation studies had insufficient data for the meta-analysis [32,34,36,37,46–52]. Among these, the reported sensitivities were above 90% only in the studies by Qiu et al. and Ölmez et al. [36,37]. Additionally, except the models for anterior and posterior horns and the body of the meniscus in the study by Tack and colleagues [34], axial MRI in Kara and colleagues’ study [46], and the study by Sharma et al. [52], all of the reported AUCs in the mentioned studies were high (≥0.9) [32,34,36].

Few studies concentrate on AI performance in detecting meniscus damage compared to other pathologies. A systematic review by Kunze et al. [54] identified five studies investigating meniscus tears. For AI algorithms, they reported an AUC range of 0.84 to 0.91 and a prediction accuracy range of 75% to 90%. Likewise, they mentioned that AI algorithms did not outperform expert clinicians [54]. Similarly, according to our pooled results in the internal validation setting, clinicians’ diagnostic performance surpassed AI algorithms, even separately for medial and lateral menisci in subgroup analysis. In another review by Fritz et al. [55], a wide range of results were reported. The sensitivity ranged from 58% to 89%, with considerably lower sensitivity for lateral meniscus, and specificity ranged from 74% to 92% [55]. Our result also had the same pattern, demonstrating higher pooled sensitivity, specificity, and AUC values for the medial meniscus. Another point of interest in the studies was to develop an algorithm for the segmentation, classification, and diagnosis of anterior cruciate ligament (ACL) tears. In the study by Dung et al. [56], they achieved an accuracy of 92% for fully ruptured ACL. Our primary assumption is that these algorithms might be beneficial in further studies to classify and detect meniscal damages.

Because of some limitations, our results should be judged cautiously. First, although adherence to the TRIPOD checklist was acceptable, 70% of the included studies were found to be high risk, according to PROBAST. Second, subgroup and meta-regression analysis was conducted for data augmentation and transfer learning, view of imaging, and meniscus tear because of noticeable heterogeneity between the studies. Third, the publication bias analysis illustrated that internal validation studies were associated with a higher risk of publication bias. Fourth, studies externally validating clinicians were not found, which complicates the interpretation of external validation results due to the need for a comparator. On the other hand, none of the included studies reported data on the AI-aided diagnostic performance of the clinicians. Correspondingly, further studies are needed in this field to estimate the effect of implementing AI accurately in detecting meniscal damage. Last but not least, the terminology of meniscal damage comprises various subtypes, including traumatic, degenerative, or mixed tears that can be partial or complete [57]. Meniscal displacement, fragment dislocation, disinsertion, etc., are other causes of meniscal damage [57]. In line with this, a high level of between-study heterogeneity was found in the terminology used to define the meniscal damage subtypes. Almost half of the included studies did not report for meniscal damage subtype [2,9,29,31,34,36–39,41,43,44], and even the ones did not report the exact category [30,32,33]. Therefore, performing a subgroup analysis based on the meniscal damage subtype was impossible. In addition, the between-study heterogeneity was even beyond this. Nine studies [29,30,32,34,35,37,38,41,43] tested their algorithm only on sagittal planes of MRI, which can lead to a misinterpretation of the result. Another possible obstacle in interpreting our results is false positive findings due to conditions such as meniscal ossicles [58]. However, the details of the reported characteristics of meniscal damage are mentioned in S4 Table.

## Conclusion

To conclude, using AI as a diagnostic tool is burgeoning, especially in image-based diagnoses. The results of this study imply the lower diagnostic performance of AI-based algorithms in knee meniscal injuries compared with radiologists. Future studies providing data on the performance of AI algorithms in detecting various meniscal damage subtypes are warranted to shed light on the exact applicability of AI-based algorithms in real-world clinical settings. Another interest of future studies could be determining the validity of AI-based algorithms in identifying meniscal lesions when acquiring surgical treatments.

## Supporting information

S1 Table Search strategy. (DOCX)

S2 Table TRIPOD modifications. (DOCX)

S3 Table PROBAST modifications. (DOCX)

S4 Table AI algorithm characteristics. (DOCX)

S5 Table Internal validation results for algorithms and clinicians. (DOCX)

S6 Table External validation results for algorithms and clinicians. (DOCX)

S7 Table Meta-regression, AI on internal validation. (DOCX)

S8 Table Meta-regression, AI on internal validation lateral meniscus. (DOCX)

S9 Table Meta-regression, AI on internal validation medial meniscus. (DOCX)

S10 Table Meta-regression, AI on clinicians internal validation. (DOCX)

S11 Table Meta-regression, AI on external validation. (DOCX)

S12 Table Publication bias. (DOCX)

S1 File All studies found in search. (XLSX)

S2 File PRISMA_2020_checklist (2). (DOCX)

S3 File Raw data. (XLSX)
