# Supplementary material for: Diagnosis of knee meniscal injuries using artificial intelligence: A systematic review and meta-analysis of diagnostic performance
Source: PLoS One. 2025 Jun 24;20(6):e0326339. doi: 10.1371/journal.pone.0326339 (PMC12186967; doi:10.1371/journal.pone.0326339)
Supplement: S1 Table — (DOCX) [file pone.0326339.s001.docx]

**Table S1.** Search strategy

| **Database** | **Search string** | **Number of results** |
| --- | --- | --- |
| **Scopus** | (“semi-lunar cartilage” OR “semi lunar cartilage” OR “tibial menisc*” OR “knee menisc*” OR “menisc*”) AND (“Defect” OR “lesion” OR “knee pain” OR “tear” OR “injury” OR “comprehensive knee assessment”) AND ("artificial intelligence" OR "machine learning" OR "deep learning" OR "neural network*" OR "computer vision") | 1381 |
| **PubMed** | (“semi-lunar cartilage” OR “semi lunar cartilage” OR “tibial menisc*” OR “knee menisc*” OR “menisc*”) AND ("artificial intelligence" OR "machine learning" OR "deep learning" OR "neural network*" OR "computer vision") | 81 |
| **EBSCO CINAHL** | (“semi-lunar cartilage” OR “semi lunar cartilage” OR “tibial menisc*” OR “knee menisc*” OR “menisc*”) AND ("artificial intelligence" OR "machine learning" OR "deep learning" OR "neural network*" OR "computer vision") | 64 |
| **WEB OF SCIENCE** | (“semi-lunar cartilage” OR “semi lunar cartilage” OR “tibial menisc*” OR “knee menisc*” OR “menisc*”) AND ("artificial intelligence" OR "machine learning" OR "deep learning" OR "neural network*" OR "computer vision") | 105 |
| **IEEE XPLORE** | (“semi-lunar cartilage” OR “semi lunar cartilage” OR “tibial menisc*” OR “knee menisc*” OR “menisc*”) AND ("artificial intelligence" OR "machine learning" OR "deep learning" OR "neural network*" OR "computer vision") | 19 |
| **COCHRANE CENTRAL (TRAILS AND REVIEWS)** | (“semi-lunar cartilage” OR “semi lunar cartilage” OR “tibial menisc*” OR “knee menisc*” OR “menisc*”) AND ("artificial intelligence" OR "machine learning" OR "deep learning" OR "neural network*" OR "computer vision") | 492 |

Results for (“semi-lunar cartilage” OR “semi lunar cartilage” OR “tibial menisc*” OR “knee menisc*” OR “menisc*”) in Scopus: 89120

Results for (“Defect” OR “lesion” OR “knee pain” OR “tear” OR “injury” OR “comprehensive knee assessment”) in Scopus: 8686045

Results for ("artificial intelligence" OR "machine learning" OR "deep learning" OR "neural network*" OR "computer vision") in Scopus: 4830045

Results for (“semi-lunar cartilage” OR “semi lunar cartilage” OR “tibial menisc*” OR “knee menisc*” OR “menisc*”) in PubMed: 20749

Results for ("artificial intelligence" OR "machine learning" OR "deep learning" OR "neural network*" OR "computer vision") in PubMed: 186640
