# Supplementary material for: Diagnosis of knee meniscal injuries using artificial intelligence: A systematic review and meta-analysis of diagnostic performance
Source: PLoS One. 2025 Jun 24;20(6):e0326339. doi: 10.1371/journal.pone.0326339 (PMC12186967; doi:10.1371/journal.pone.0326339)
Supplement: S2 Table — (DOCX) [file pone.0326339.s002.docx]

Table S2. TRIPOD modifications

| **TRIPOD item** | **Modifications** |
| --- | --- |
| 4a | Consider “+” if all of the training, testing, and validation sets are defined. |
| 4b | Consider irrelevant in the absence of a follow-up period. |
| 5b | Consider “+” if the eligibility criteria is described for images instead of participants. |
| 5c | Not relevant |
| 6a | Consider “+” in the presence of a reference standard. |
| 6b | Not relevant |
| 7a/b | Not relevant |
| 9 | Consider N/A if participants’ clinical information is not included in the study. |
| 10a | Not relevant |
| 10b | Consider “+” in the presence of a precise description for AI algorithm’s building process. |
| 10c | Not relevant |
| 11 | Not relevant |
| 12 | Consider “+” in external validation studies with a clear description of external validation setting, otherwise N/A |
| 13a | Consider “+” in the presence of a flow-chart of the images |
| 13b/c | Consider “+” if age and sex of the participants in the dataset are reported |
| 14a | Consider “+” if the number of disease+ participants is included |
| 14b | Not relevant |
| 15a | Not relevant |
| 15b | Consider “+” if a mean for interpretation is provided in the model |
| 19a | Consider “+” if a plausible comparison between similar studies is included |
| 21 | Consider “+” if it is mentioned that the supplementary data is accessible |
