# Supplementary material for: Diagnosis of knee meniscal injuries using artificial intelligence: A systematic review and meta-analysis of diagnostic performance
Source: PLoS One. 2025 Jun 24;20(6):e0326339. doi: 10.1371/journal.pone.0326339 (PMC12186967; doi:10.1371/journal.pone.0326339)
Supplement: S3 Table — (DOCX) [file pone.0326339.s003.docx]

Table S3. PROBAST modifications

| **PROBAST item** | **Modifications** |
| --- | --- |
| Domain 1: Participants | Score based on data on images instead of participants |
| Domain 2: Predictors | N/A |
| Domain 3: Outcomes | 3.3, 3.5, and 3.6 are N/A |
| Domain 4: Analysis | 4.2 – 4.6, 4.8, and 4.9 are N/A |
| Domain 5: Overall | - |
