# Supplementary material for: Diagnosis of knee meniscal injuries using artificial intelligence: A systematic review and meta-analysis of diagnostic performance
Source: PLoS One. 2025 Jun 24;20(6):e0326339. doi: 10.1371/journal.pone.0326339 (PMC12186967; doi:10.1371/journal.pone.0326339)
Supplement: S4 Table — (DOCX) [file pone.0326339.s004.docx]

**Table S4. AI algorithm characteristics**

| First author | Year | Study design | Inclusion criteria | Exclusion criteria | Number of participants represented by the training data | Disease+ participants% | Male% of participants | Mean age of participants  (SD) | Neural Network Algorithm | Neural Network Architecture | Tesla | Dimension | Tear | Subtype | validation method | Used data augmentation | Used transfer learning |
| --- | --- | --- | --- | --- | --- | --- | --- | --- | --- | --- | --- | --- | --- | --- | --- | --- | --- |
|  | Internal Validation | | | | | | | | | | | | | | | | |
| Bharath Ramakrishna | 2008 | Case control | NR^[[1]](#footnote-1)^ | NR | 40 | 42.5 | 47.5 | Male ages range 15–61, Females ages range 30–73 | novel computer-aided detection (CAD) | NR | 1.5 | 512x512 | Yes | Simple and complex meniscal tears, meniscal fragment, degenerative tear | Training set | No | No |
| M. H. Fazel Zarandi | 2016 | Case control | NR | NR | 28 | 74.78 | 64.28 | male with a range of 25 to 64 years old, female with a range of 44 to 78 years old | type-2 λ-enhancement algorithm, PNN | NR | NR | NR | Yes | NR | random split sample validation | No | Yes |
| Ahmet Saygılı | 2018 | Case control | NR | NR | 29 | 100 | 55 | NR | KNN^[[2]](#footnote-2)^, ELM, SVM^[[3]](#footnote-3)^ | NR | 3 | 384 × 384 | Yes | Horizontal tears, Vertical tears, Partial Maceration | random split sample validation | No | No |
| Nicholas Bien | 2018 | Case control | NR | NR | 1312 | 80.6 | 58.5 | 38 | CNN^[[4]](#footnote-4)^ | MRNet, AlexNet | 1.5 | 256 × 256 | Yes | NR | stratified split sampling | No | Yes |
| V. Couteaux | 2019 | Case control | NR | NR | NR | NR | NR | NR | R-CNN | ResNet | NR | 256 × 256 | Yes | Vertical tear, horizontal tear | random split sample validation | No | No |
| Benjamin Fritz | 2020 | Case control | (1) MRI of the knee joint performed at our institution on a clinical 1.5 Tesla or 3 Tesla clinical whole body MRI system using our standard protocols for evaluation of knee pain (2) arthroscopic knee surgery performed at our institution by a specialized knee surgeon, at a time interval of less than 3 months after the knee MRI (3) signed informed consent for retrospective data analysis. | 1- previous knee surgery 2- impaired image quality due to motion | 100 | 69 | 54 | 39.9(14.3) | DCNN | NR | 1.5 or 3 | 336 × 448 | Yes | Horizontal tears | Validation set | No | No |
| Emre ÖLMEZ | 2020 | Case control | NR | NR | NR | NR | NR | NR | R-CNN | ConvNet | 1.5 | 65×78 | No | NR | random split sample validation | No | Yes |
| Alexander Tack | 2021 | Case control | NR | NR | NR | DESS, Medial meniscus: 40  DESS, Lateral meniscus: 20  IW TSE, Medial meniscus: 40.1  IW TSE, Lateral meniscus: 20.1 | 37.93 | 61.88(8.87) | CNN | ResNet, nnU-net | NR | DESS: 384 × 384, IW TSE : 442 × 448 | Yes | NR | Random split sample validation | Yes | No |
| Bruno Astuto | 2021 | Case control | NR | concurrent use of an investigational drug, fracture or surgical intervention in the study knee, and any contraindications to MRI | 294 | NR | 47.9 | 43(15) | CNN | 3D V-Net | 3 | 280x280 | Yes | Nondisplaced or Displace Tear, Partial Resection, Complete Maceration | stratified split sampling | Yes | No |
| Xubin Qiu | 2021 | Case control | NR | NR | 205 | 80.13 | 54.63 | 37.52(2.86) | CNNf | VGG-16 | 3 | 512×512 | Yes | NR | training set | No | No |
| Ali Can Kara 2021 | 2021 | Case control | NR | NR | 130 | NR | NR | NR | CNN | ResNet50 | NR | 256×256 | Yes | NR | random split sample validation | Yes | Yes |
| Hyunkwang Shin | 2022 | Case control | NR | NR | NR | 57.15 | 69.56 | 38.7(16.5) | CNN | DenseNet | 1.5 | 224×224 | Yes | NR | Random split sample validation | No | No |
| Jie Li | 2022 | Case control | The images without motion artifacts or any other magnetic artifacts were included. | NR | 1104 | NR | NR | NR | R-CNN | ResNet50, 101-FPN | 1.5(90 patients of external test used 1.5 tesla), 3(internal set and 90 patients of external set used 3 tesla) | 1188x1372 pixels | Yes | Degenerative | validation set | Yes | No |
| Yuan‑Zhe Li | 2022 | Case control | Knee MRI before Arthroscopic surgery/ Arthroscopic knee surgery/ Signed informed consent | Unavailable or incomplete clinical or MRI information; poor quality MRI images with a low signal-to-noise ratio (SNR) | 533 | NR | 63.03 | 51.3(10.5) | 3D-Mask RCNN | 3D-UNET | 3 | 256×256 | Yes |  | stratified split sampling | No | No |
| Truong Nguyen Khanh Hung | 2022 | Case control | patients with arthroscopically confirmed meniscus injury and patients who underwent MRI scanning before treatment. | treatment before MRI scanning; unavailable or incomplete clinical or MRI information; poor MRI image quality with low signal-to-noise ratio (SNR); pregnant women and lactating. | 152 | NR | 63.81 | 52.4(11) | CNN | YOLOv4, DarkNet53 | 3 | 512×512( for internal test set), 256×256(for external test set) | Yes | NR | random split sample validation | No | No |
| Yi-Ting Chou | 2022 | Case control | NR | Having meniscus surgical history | 704 | NR | 72.28 | 43.8 | CNN | Scaled-YOLOv4, EfficientNet-B7 | NR | 512×512 | Yes | NR | Cross validation | Yes | No |
| Yi Wang | 2022 | Case control | NR | NR | 768 | 52.03 | 62.9 | 29.7 | minimum redundancy maximum relevance (mRMR) algorithm | NR | 3 | 256 × 256 | Yes | Degenerative | Cross validation | No | No |
| Shilpa Sharma | 2022 | Case control | NR | NR | 1088 | 37.1 | NR | NR | CNN | ResNet50 | NR | 256×256 | Yes | NR | random split sample validation | No | Yes |
| Yingkai Ma | 2023 | Case control | 1. Age > 16 years old, 2. Patients were diagnosed with meniscus injury. | 1. Patients with knee MRI images with pixels too fuzzy to detect. 2. Patients with knee ligament injuries or other diseases. | 1396 | 48.71 | NR | NR | CNN | ATM-THREE, ATM-SECOND, C-PCNN, EfficientNet B0, EfficientNet Bl , MobileNet, ResNet34, ResNet50, VGG | NR | NR | Yes | horizontal tears | cross validation | No | No |
| Anita Thengade | 2023 | Case control | NR | NR | 1370 | 37.08 | NR | NR | DCNN | NASNet Mobile, NASNet Large, NASNet Large (Middle 9 slices), ResNet50, ResNet50 (Middle 9 slices), DCNN with Residual Learning | NR | 256 × 256 | Yes | NR | random split sample validation | No | Yes |
| Massimiliano Mangone | 2023 | Case control | NR | 1. Patients with previous surgical interventions (i.e., ACL reconstruction, partial meniscectomy, etc.) 2. The interobserver rate on the evaluation of the cases by the radiologists was very low, consisting of only three cases. | 528 | 33.52 | 57.19 | 44.93 | CNN | AlexNet | NR | 224 × 224 | Yes | broken, radial, longitudinal, or fracture lines present in at least three slices or morphologic deformity | random split sample validation | No | No |
| FATMA HARMAN | 2023 | Case control | NR | NR | NR | NR | NR | NR | NR | UNET, Invertible Recurrent Inference Machine (iRIM), E2E VarNET, | 1.5, 3 | NR | Yes | NR | cross validation | Yes | No |
| Erdal Güngör | 2024 | Case control | Patients aged between 30 and 45 years who underwent MRI examinations from 2022–2024 at their hospital. | Patients with a history of knee surgery were excluded. | 642 | 67.44 | 56.9 | 36.2 | NR | EfficientNetV2, YOLOv8 | NR | 480 × 480 | Yes | NR | random split sample validation | Yes | No |
| Kexin Jiang | 2024 | Case control | NR | 1. Knees from other project except POMA project and FNIH project 2. Knees without available MOAKS measurement. | 4796 | 26.93 | 38.09 | 61.65 | CNN | ResNet26 | 3 | 224×224 | Yes | NR | random split sample validation | Yes | No |
|  | External Validation | | | | | | | | | | | | | | | | |
| Cemal Kose | 2007 | Case control | NR | NR | 54 | 42.8 | NR | NR | Ostu adaptive threshold | Simple histogram based, statistical segmenta tion methods | NR | NR | Yes | NR | Training set | No | No |
| Valentina Pedoia | 2018 | Case control | NR | NR | 302 | NR | 48 | 42.79(14.75) | 3D-CNN, RF, SVM, logistic regression, decision trees | 2D U-Net | NR | 512×512 | No | Displaced or complex tears without deformity, maceration of the meniscus | random split sample validation | Yes | No |
| V. Roblot | 2019 | Case control | normal menisci or those with abnormal grade 3 high meniscal signal intensity according to Stoller’s classification (abnormal hyperintensity that extends to at least one articular surface, superior or inferior) were included. | NR | NR | 13 | NR | NR | Fast R-CNN | NR | NR | 256 × 256 | Yes | NR | random split sample validation | Yes | No |
| B. Rizk | 2021 | Case control | NR | 1- Patients under the age of 16 (N = 309) 2-Patients with a known past knee surgical history (N = 2189). | 7903 | NR | 51.9 | 43.6 | CNN | MRNet | 1, 1.5, 3 | 64×64 | Yes | NR | Cross validation | No | No |
| Jie Li | 2022 | Case control | The images without motion artifacts or any other magnetic artifacts were included. | NR | 1104 | NR | NR | NR | R-CNN | ResNet50, 101-FPN | 1.5(90 patients of external test used 1.5 tesla), 3(internal set and 90 patients of external set used 3 tesla) | 1188x1372 pixels | Yes | Degenerative | validation set | Yes | No |
| Truong Nguyen Khanh Hung | 2022 | Case control | patients with arthroscopically confirmed meniscus injury and patients who underwent MRI scanning before treatment. | treatment before MRI scanning; unavailable or incomplete clinical or MRI information; poor MRI image quality with low signal-to-noise ratio (SNR); pregnant women and lactating. | 152 | NR | 63.81 | 52.4(11) | CNN | YOLOv4, DarkNet53 | 3 | 512×512( for internal test set), 256×256(for external test set) | Yes | NR | random split sample validation | No | No |

1. Not reported (NR) [↑](#footnote-ref-1)
2. k-nearest neighbors’ algorithm (KNN) [↑](#footnote-ref-2)
3. Support vector machine (SVM) [↑](#footnote-ref-3)
4. Convolutional neural network (CNN) [↑](#footnote-ref-4)
