# Supplementary material for: Diagnosis of knee meniscal injuries using artificial intelligence: A systematic review and meta-analysis of diagnostic performance
Source: PLoS One. 2025 Jun 24;20(6):e0326339. doi: 10.1371/journal.pone.0326339 (PMC12186967; doi:10.1371/journal.pone.0326339)
Supplement: S5 Table — (DOCX) [file pone.0326339.s005.docx]

Table S5

Internal Validation Results for Algorithms and Clinicians

| First author and year | Algorithm results: sensitivity, specificity | Algorithm results: other metrics | Clinician results: sensitivity, specificity | Clinician results: other metrics |
| --- | --- | --- | --- | --- |
| Bharath Ramakrishna 2008 | Meniscus: 0.8387, 0.7519  Medial anterior meniscus: 67, 84  Medial posterior meniscus: 94, 71  Lateral anterior meniscus: 75, 70  Lateral posterior meniscus: 75, 75 | Ppv^[[1]](#footnote-1)^, npv^[[2]](#footnote-2)^, accuracy, f1:  Meniscus: 0.45, 0.95, 0.77, 0.58  Medial anterior meniscus: 0.25, 0.97, 0.82, 0.36  Medial posterior meniscus: 0.68, 0.94, 0.8, 0.79  Lateral anterior meniscus: 0.21, 0.96, 0.7, 0.33  Lateral posterior meniscus: 0.43, 0.92, 0.75, 0.55 | Average: 0.77, 0.81  Radiologist 1 medial anterior meniscus: 0.67, 0.89  Radiologist 1 medial posterior meniscus: 0.94, 0.63  Radiologist 1 Lateral anterior meniscus: 0.25, 0.81  Radiologist 1 Lateral posterior meniscus: 0.63, 0.75  Radiologist 2 medial anterior meniscus: 0.33, 0.92  Radiologist 2 medial posterior meniscus: 0.94, 0.83  Radiologist 2 Lateral anterior meniscus: 0.5, 0.81  Radiologist 2 Lateral posterior meniscus: 0.88, 0.81 | Ppv, npv, accuracy, f1:  Average: 0.5, 0.94, 0.81, 0.61  Radiologist 1 medial anterior meniscus: 0.33, 0.97, 0.87, 0.44  Radiologist 1 medial posterior meniscus: 0.62, 0.94, 0.75, 0.75  Radiologist 1 Lateral anterior meniscus: 0.12, 0.81, 0.75, 0.17  Radiologist 1 Lateral posterior meniscus: 0.39, 0.89, 0.72, 0.48  Radiologist 2 medial anterior meniscus: 0.25, 0.94, 0.87, 0.28  Radiologist 2 medial posterior meniscus: 0.79, 0.95, 0.87, 0.86  Radiologist 2 Lateral anterior meniscus: 0.22, 0.93, 0.77, 0.31  Radiologist 2 Lateral posterior meniscus: 0.54, 0.96, 0.82, 0.67 |
| M. H. Fazel Zarandi 2016 | 0 and 1 mode: 0.92  Binary Classification: 0.83 | Ppv, accuracy, f1:  0 and 1 mode: 0.98, 0.9, 0.95  Binary Classification: 0.93, 0.78, 0.88 |  |  |
| Ahmet Saygılı 2018 | **Binary Classification**  (KNN, ELM, SVM)- Meniscus: 0.95, 0.86  KNN- (Medial Meniscus- Anterior Horn): 0.97, 0.5  KNN- (Medial Meniscus- Body): 0.79, 0.86  KNN- (Medial Meniscus- Posterior Horn): 0.71, 0.86  ELM- (Medial Meniscus- Anterior Horn): 1, 0.55  ELM- (Medial Meniscus- Body): 0.81, 0.89  ELM- (Medial Meniscus- Posterior Horn): 0.74, 0.89  SVM- (Medial Meniscus- Anterior Horn): 0.97, 0.5  SVM- (Medial Meniscus- Body): 0.77, 0.84  SVM- (Medial Meniscus- Posterior Horn): 0.7, 0.85  KNN- (Lateral Meniscus- Anterior Horn): 0.98, 0.51  KNN- (Lateral Meniscus- Body): 0.92, 0.45  KNN- (Lateral Meniscus- Posterior Horn): 0.97, 0.5  ELM- (Lateral Meniscus- Anterior Horn): 0.99, 0.55  ELM- (Lateral Meniscus- Body): 0.99, 0.54  ELM- (Lateral Meniscus- Posterior Horn): 0.99, 0.55  SVM- (Lateral Meniscus- Anterior Horn): 0.98,0.5  SVM- (Lateral Meniscus- Body): 0.91, 0.42  SVM- (Lateral Meniscus- Posterior Horn): 0.97, 0.46  **Multiclass Classification:**  KNN- (Medial Meniscus- Anterior Horn): 0.74, 0.49  KNN- (Medial Meniscus- Body): 0.76, 0.78  KNN- (Medial Meniscus- Posterior Horn): 0.70, 0.90  ELM- (Medial Meniscus- Anterior Horn): 0.80, 0.53  ELM- (Medial Meniscus- Body): 0.86,0.95  ELM- (Medial Meniscus- Posterior Horn): 0.83, 0.95  SVM- (Medial Meniscus- Anterior Horn): 0.75, 0.47  SVM- (Medial Meniscus- Body): 0.74, 0.82  SVM- (Medial Meniscus- Posterior Horn): 0.74, 0.92  KNN- (Lateral Meniscus- Anterior Horn): 0.93, 0.46  KNN- (Lateral Meniscus- Body): 0.91, 0.43  KNN- (Lateral Meniscus- Posterior Horn): 0.92, 0.49  ELM- (Lateral Meniscus- Anterior Horn): 0.96, 0.5  ELM- (Lateral Meniscus- Body): 0.97, 0.54  ELM- (Lateral Meniscus- Posterior Horn): 0.99, 0.52  SVM- (Lateral Meniscus- Anterior Horn): 0.93, 0.46  SVM- (Lateral Meniscus- Body): 0.89, 0.41  SVM- (Lateral Meniscus- Posterior Horn): 0.93, 0.45 | Ppv, npv, accuracy, f1:  **Binary Classification**  (KNN, ELM, SVM)- Meniscus: 0.71, 0.98, 0.85, 0.81  KNN- (Medial Meniscus- Anterior Horn): 0.13, 0.99, 0.90, 0.22  KNN- (Medial Meniscus- Body): 0.86, 0.79, 0.82, 0.82  KNN- (Medial Meniscus- Posterior Horn): 0.93, 0.53, 0.82, 0.81  ELM- (Medial Meniscus- Anterior Horn): 0.14, 1, 1, 0.25  ELM- (Medial Meniscus- Body): 0.9, 0.82, 0.81, 0.85  ELM- (Medial Meniscus- Posterior Horn): 0.95, 0.57, 0.74, 0.83  SVM- (Medial Meniscus- Anterior Horn): 0.12, 0.99, 0.90, 0.22  SVM- (Medial Meniscus- Body): 0.84, 0.77, 0.80, 0.8  SVM- (Medial Meniscus- Posterior Horn): 0.92, 0.52, 0.90, 0.22  KNN- (Lateral Meniscus- Anterior Horn): 0.19, 0.99, 0.80, 0.8  KNN- (Lateral Meniscus- Body): 0.06, 0.92, 0.8, 0.8  KNN- (Lateral Meniscus- Posterior Horn): 0.1, 0.89, 0.91, 0.32  ELM- (Lateral Meniscus- Anterior Horn): 0.2, 0.99, 0.85, 0.1  ELM- (Lateral Meniscus- Body): 0.08, 0.94, 0.9, 0.17  ELM- (Lateral Meniscus- Posterior Horn): 0.12, 0.91, 0.93, 0.2  SVM- (Lateral Meniscus- Anterior Horn): 0.18, 0.99, 0.93, 0.31  SVM- (Lateral Meniscus- Body): 0.05, 0.91, 0.83, 0.1  SVM- (Lateral Meniscus- Posterior Horn): 0.1, 0.88, 0.88, 0.15  **Multiclass Classification:**  KNN- (Medial Meniscus- Anterior Horn): 0.1, 0.96, 0.73, 0.17  KNN- (Medial Meniscus- Body): 0.8, 0.76, 0.77, 0.78  KNN- (Medial Meniscus- Posterior Horn): 0.95, 0.53, 0.81, 0.81  ELM- (Medial Meniscus- Anterior Horn): 0.11, 0.97, 0.77, 0.2  ELM- (Medial Meniscus- Body): 0.95, 0.86, 0.85, 0.9  ELM- (Medial Meniscus- Posterior Horn): 0.98, 0.68, 0.85, 0.9  SVM- (Medial Meniscus- Anterior Horn): 0.1, 0.96,0.71, 0.17  SVM- (Medial Meniscus- Body): 0.81, 0.75, 0.76, 0.78  SVM- (Medial Meniscus- Posterior Horn): 0.96, 0.57, 0.84, 0.84  KNN- (Lateral Meniscus- Anterior Horn): 0.17, 0.99, 0.86, 0.29  KNN- (Lateral Meniscus- Body): 0.05, 0.9, 0.83, 0.1  KNN- (Lateral Meniscus- Posterior Horn): 0.1, 0.89, 0.84, 0.16  ELM- (Lateral Meniscus- Anterior Horn): 0.18, 0.99, 0.89, 0.31  ELM- (Lateral Meniscus- Body): 0.08, 0.94, 0.88, 0.14  ELM- (Lateral Meniscus- Posterior Horn): 0.11, 0.90, 0.89, 0.2  SVM- (Lateral Meniscus- Anterior Horn): 0.16, 0.99, 0.86, 0.3  SVM- (Lateral Meniscus- Body): 0.05, 0.90, 0.82, 0.1  SVM- (Lateral Meniscus- Posterior Horn): 0.08, 0.87, 0.84, 0.14 |  |  |
| Nicholas Bien 2018 |  |  | 0.82, 0.88 | Ppv, npv, accuracy, f1:  0.87, 0.83, 0.85{0.82, 0.87}, 0.84 |
| V. Couteaux 2019 |  | Auc^[[3]](#footnote-3)^:  0.91 |  |  |
| Benjamin Fritz 2020 | Medial meniscus: 0.93, 0.88  Lateral meniscus: 0.71, 0.92  Medial and lateral meniscus: 0.94, 0.87 | Ppv, npv, auc, accuracy, f1:  Medial meniscus: 0.91, 0.90, 0.88,  0.86, 0.92  Lateral meniscus: 0.74, 0.91, 0.78, 0.84, 0.72  Medial and lateral meniscus: 0.50, 0.99, 0.96, 0.9, 0.65 | Reader1  Medial meniscus:0.93, 0.91  Lateral meniscus:0.71, 0.95  Medial and lateral meniscus: 0.94, 0.87  Reader2  Medial meniscus: 0.96, 0.86  Lateral meniscus: 0.67, 0.99  Medial and lateral meniscus: 0.94, 0.94 | Ppv, npv, auc, accuracy, f1:  Reader1  Medial meniscus: 0.93, 0.91, 0.91[0.85-0.96], 0.92, 0.93  Lateral meniscus: 0.81, 0.91, 0.83[0.74-0.90], 0.89, 0.75  Medial and lateral meniscus: 0.50, 0.99, 0.91[0.83-0.96], 0.92, 0.65  Reader2  Medial meniscus: 0.90, 0.95, 0.91[0.84-0.96], 0.92, 0.93  Lateral meniscus: 0.94, 0.90, 0.83[0.74-0.89], 0.91, 0.78  Medial and lateral meniscus:0.67, 0.99, 0.94[0.87-0.98], 0.94, 0.78 |
| Emre ÖLMEZ 2020 | Data set1: 0.947, NR  Data set2: NR, NR | Accuracy:  Data set1: NR  Data set2: 0.93 |  |  |
| Alexander Tack 2021 |  | Auc:  DESS:  medial meniscal (ant horn): 0.94  medial meniscal(body): 0.93  medial meniscal (post horn): 0.93  lateral meniscal (ant horn): 0.96  lateral meniscal(body): 0.94  lateral meniscal (post horn): 0.91  IW TSE:  medial meniscal (ant horn): 0.84  medial meniscal(body): 0.88  medial meniscal (post horn): 0.86  lateral meniscal (ant horn): 0.95  lateral meniscal(body): 0.91  lateral meniscal (post horn): 0.9 |  |  |
| Bruno Astuto 2021 | 0.85, 0.85 | Ppv ,npv, auc, accuracy, f1:  0.87, 0.83, 0.93(0.02), 0.85, 0.86 |  |  |
| Xubin Qiu 2021 | 0.91, 0.95 | Auc, accuracy:  0.97, 0.94 |  |  |
| Ali Can Kara 2021 | Sagittal view: 0.56, 0.92  Coronal view: 0.71, 0.78  Axial view: 0.71, 0.67 | Auc, accuracy:  Sagittal view: 0.8, 0.77  Coronal view: 0.94, 0.75  Axial view: 0.71, 0.69 |  |  |
| Hyunkwang Shin 2022 | Medial meniscus tear: 0.83, 0.87  Lateral meniscus tear: 0.68, 0.85  Medial and lateral meniscus tear: 0.78, 0.93 | Ppv, npv, auc, accuracy, f1:  Medial meniscus tear: 0.83, 0.85, 0.89[0.85-0.93], 0.85, 0.84  Lateral meniscus tear: 0.63, 0.88, 0.82[0.74-0.89], 0.80, 0.65  Medial and lateral meniscus tear: 0.55, 0.98, 0.92[0.86-0.99], 0.92, 0.65 |  |  |
| Jie Li 2022 | Bbox: 0.84, 0.99  Mask:0.74, 0.99 | Ppv, npv, accuracy, f1:  Bbox: 0.99, 0.88, 0.92, 0.91  Mask: 0.99, 0.82, 0.88, 0.85 |  |  |
| Yuan‑Zhe Li 2022 | 0.941, 0.785 | Ppv, npv, auc, accuracy, f1:  0.86, 0.89, 0.91, 0.924, 0.9 | 0.889, 0.754 | Ppv, npv, auc, accuracy, f1:  0.84, 0.81, 0.83, 0.835, 0.87 |
| Truong Nguyen Khanh Hung 2022 | Data set1: 0.948, 0.961  Data set2: 0.986, 0.92 | Ppv, npv, accuracy, f1:  Data set1: 0.96, 0.94, 0.95, 0.96  Data set2: 0.94, 0.96, 0.95, 0.96 |  |  |
| Yi-Ting Chou 2022 | Ant Medial meniscus: 0.85, 0.97  Ant lateral meniscus: 0.88, 0.98  Post Medial meniscus: 0.91, 0.97  Post lateral meniscus: 0.84, 0.98  Meniscus (sagittal view): NR, NR  Meniscus (coronal view):NR, NR | Ppv, npv, auc, accuracy, f1:  Ant Medial meniscus: 0.85, 0.97, 0.97, 0.95, 0.85  Ant lateral meniscus: 0.88, 0.98, 0.97, 0.97, 0.88  Post Medial meniscus: 0.87, 0.98, 0.89, 0.962, 0.89  Post lateral meniscus: 0.93, 0.95, 0.98, 0.94, 0.89  Meniscus (sagittal view): NR, NR, 0.98, NR, 0.88  Meniscus (coronal view): NR, NR, 0.97, NR , 0.74 |  |  |
| Yi Wang 2022 | Coronal and sagittal view: 0.88, 0.895  Sagittal view: 0.871, 0.855  Coronal view: 0.847, 0.853 | Auc, accuracy:  Coronal and sagittal view: 0.92(0.83-0.996), 0.891  Sagittal view: 0.876(0.875-0.984), 0.862  Coronal view: 0.851(0.834-0.921), 0.879 |  |  |
| Shilpa Sharma 2022 |  | Auc, accuracy:  0.82, 0.79 |  |  |
| Yingkai Ma 2023 | ATM-THREE: 0.82, 0.87  ATM-SECOND: 0.82, 0.88  C-PCNN:0.8. 0.91  EfficientNet B0:0.85, 0.68  EfficientNet Bl:0.85, 0.68  MobileNet: 0.78, 0.77  ResNet34:0.84, 0.77  ResNet50: 0.86, 0.67  VGG:0.82, 0.8 | ppv, npv, auc, accuracy, f1:  ATM-THREE: 0.86, 0.84, 0.85, 0.85, 0.84  ATM-SECOND: 0.59, 0.84, 0.85, 0.85, 0.84  C-PCNN: 0.9, 0.83, 0.86, 0.86, 0.85  EfficientNet B0: 0.72, 0.83, 0.78, 0.76, 0.78  EfficientNet Bl: 0.71, 0.83, 0.79, 0.76, 0.78  MobileNet: 0.76, 0.79, 0.74, 0.78, 0.78  ResNet34: 0.78, 0.84, 0.79, 0.81, 0.81  ResNet50: 0.85, 0.33, 0.83, 0.79, 0.8  VGG: 0.79, 0.83, 0.82, 0.81, 0.81 |  | PPV:  Attending doctor 1: 0.90  Attending doctor 2: 0.91  Attending doctor 3: 0.9  Chief 1: 0.975  Chief 2: 0.955  Attending doctor 1+ CNN: 0.96  Attending doctor2+ CNN: 0.97  Attending doctor 3+ CNN: 0.97 |
| Anita Thengade 2023 |  | Accuracy:  Sagittal:  NASNet Mobile:0.61  NASNet Large: 0.60  NASNet Large (Middle 9 slices): 0.65  ResNet50: 0.70  ResNet50 (Middle 9 slices): 0.63  DCNN with Residual Learning: 0.82  Axial:  NASNet Mobile: 0.68  NASNet Large: 0.63  NASNet Large (Middle 9 slices): 0.58  ResNet50: 0.74  ResNet50 (Middle 9 slices): 0.84  DCNN with Residual Learning: 0.86  Coronal:  NASNet Mobile: 0.49  NASNet Large: 0.60  NASNet Large (Middle 9 slices): 0.59  ResNet50: 0.73  ResNet50 (Middle 9 slices): 0.62  DCNN with Residual Learning: 0.84 |  |  |
| Massimiliano Mangone 2023 | 0.75, 0.92 | Accuracy:  0.84 |  |  |
| FATMA HARMAN 2023 | 4 fold accelerations  UNET: 0.48, NR  iRIM: 0.52, NR  E2E VarNET: 0.56, NR  8 fold accelerations  UNET: 0.49, NR  iRIM: 0.5, NR  E2E VarNET: 0.57, NR | Ppv:  4 fold accelerations  UNET: 0.82  iRIM: 0.87  E2E VarNET: 0.87  8 fold accelerations  UNET: 0.8  iRIM: 0.8  E2E VarNET: 0.87 |  |  |
| Erdal Güngör 2024 |  | Auc, ppv:  EfficientNetV2  Sagittal: 0.97, NR  Coronal:0.98, NR  YOLOv8  Sagittal: NR, 0.98  Coronal: NR, 0.98 |  |  |
| Kexin Jiang 2024 |  | Accuracy:  Ant Medial meniscus: 0.6  Body Medial meniscus: 0.71  Post Medial meniscus: 0.79  Ant lateral meniscus: 0.64  Body lateral meniscus: 0.72  Post lateral meniscus: 0.73 |  |  |

1. Positive predictive value (PPV) [↑](#footnote-ref-1)
2. Negative predictive value (NPV) [↑](#footnote-ref-2)
3. Area under the curve (AUC) [↑](#footnote-ref-3)
