# Supplementary material for: Diagnosis of knee meniscal injuries using artificial intelligence: A systematic review and meta-analysis of diagnostic performance
Source: PLoS One. 2025 Jun 24;20(6):e0326339. doi: 10.1371/journal.pone.0326339 (PMC12186967; doi:10.1371/journal.pone.0326339)
Supplement: S6 Table — (DOCX) [file pone.0326339.s006.docx]

Table S6

External Validation Results for Algorithms and Clinicians

| First author and year | Algorithm results: sensitivity, specificity | Algorithm results: other metrics | Clinician results: sensitivity, specificity | Clinician results: other metrics |
| --- | --- | --- | --- | --- |
| Cemal Kose 2007 | 0.883, 0.957 | Ppv^[[1]](#footnote-1)^, npv^[[2]](#footnote-2)^, accuracy, f1:  0.897, 0.9502, 0.93, 0.89 |  |  |
| Valentina Pedoia 2018 | 0.82, 0.90 | Ppv, npv, auc^[[3]](#footnote-3)^, accuracy, f1:  0.53, 0.97, 0.89, 0.88, 0.64 |  |  |
| V. Roblot 2019 |  | Auc:  0.94 |  |  |
| B. Rizk 2021 | medial meniscal- first dataset: 0.89, 0.84  lateral meniscal- first dataset: 0.67, 0.88  meniscus (meniscal tear detection with finetuning)- second dataset: 0.81, 0.87  meniscus (meniscal tear detection without finetuning)- second dataset: 0.77, 0.84 | Ppv, npv, auc, accuracy, f1:  medial meniscal- first dataset: 0.88, 0.85, 0.93[0.82-0.95], 0.87, 0.88  lateral meniscal- first dataset: 0.70, 0.86, 0.84[0.78-0.89], 0.82, 0.68  meniscus (meniscal tear detection with finetuning)- second dataset: NR, NR, 0.89[0.82-0.95], 0.84, NR  meniscus (meniscal tear detection without finetuning)- second dataset: NR, NR, 0.83[0.75-0.9], 0.81, NR |  |  |
| Jie Li 2022 |  | Accuracy:  1.5 tesla: 0.92  3 tesla: 0.61 |  |  |
| Truong Nguyen Khanh Hung 2022 | 0.79, 0.78 | Ppv, npv, accuracy, f1:  0.766, 0.783, 0.788, 0.79 |  |  |

1. Positive predictive value (PPV) [↑](#footnote-ref-1)
2. Negative predictive value (NPV) [↑](#footnote-ref-2)
3. Area under the curve (AUC) [↑](#footnote-ref-3)
