# Supplementary material for: Diagnosis of knee meniscal injuries using artificial intelligence: A systematic review and meta-analysis of diagnostic performance
Source: PLoS One. 2025 Jun 24;20(6):e0326339. doi: 10.1371/journal.pone.0326339 (PMC12186967; doi:10.1371/journal.pone.0326339)
Supplement: S7 Table — (DOCX) [file pone.0326339.s007.docx]

Table S7. Meta-Regression, AI^[[1]](#footnote-1)^ on Internal Validation

| Parameter | Category | Number of studies in each category | Sensitivity[95%CI] | P-value | Specificity[95%CI] | P-value |
| --- | --- | --- | --- | --- | --- | --- |
| View | Yes | 29 | 0.83 [0.79 - 0.87] | 0.00 | 0.89 [0.85 - 0.92] | 0.83 |
|  | No | 41 | 0.80 [0.74 - 0.86] |  | 0.63 [0.56 - 0.71] |  |
| Data Augmentation | Yes | 7 | 0.85 [0.77 - 0.93] | 0.01 | 0.98 [0.96 - 1.00] | 0.09 |
|  | No | 63 | 0.81 [0.77 - 0.85] |  | 0.73 [0.68 - 0.78] |  |
| Transfer Learning | Yes | 3 | 0.83 [0.71 - 0.95] | 0.12 | 0.41 [-0.06 - 0.88] | 0.10 |
|  | No | 67 | 0.81 [0.78 - 0.85] |  | 0.79 [0.72 - 0.84] |  |

1. Artificial intelligence (AI) [↑](#footnote-ref-1)
