# Supplementary material for: Diagnosis of knee meniscal injuries using artificial intelligence: A systematic review and meta-analysis of diagnostic performance
Source: PLoS One. 2025 Jun 24;20(6):e0326339. doi: 10.1371/journal.pone.0326339 (PMC12186967; doi:10.1371/journal.pone.0326339)
Supplement: S8 Table — (DOCX) [file pone.0326339.s008.docx]

Table S8. Meta-Regression, AI^[[1]](#footnote-1)^ on Internal Validation Lateral Meniscus

| Parameter | Category | Number of studies in each category | Sensitivity[95%CI] | P-value | Specificity[95%CI] | P-value |
| --- | --- | --- | --- | --- | --- | --- |
| View | Yes | 6 | 0.72 [0.66 - 0.78] | 0.28 | 0.90 [0.87 - 0.94] | 0.01 |
|  | No | 18 | 0.67 [0.53 - 0.80] |  | 0.49 [0.42 - 0.55] |  |
| Data Augmentation | Yes | 2 | 0.86 [0.76 - 0.96] | 0.77 | 0.98 [0.96 - 1.00] | 0.00 |
|  | No | 22 | 0.67 [0.57 - 0.77] |  | 0.57 [0.49 - 0.65] |  |

1. Artificial intelligence (AI) [↑](#footnote-ref-1)
