# Supplementary material for: Diagnosis of knee meniscal injuries using artificial intelligence: A systematic review and meta-analysis of diagnostic performance
Source: PLoS One. 2025 Jun 24;20(6):e0326339. doi: 10.1371/journal.pone.0326339 (PMC12186967; doi:10.1371/journal.pone.0326339)
Supplement: S9 Table — (DOCX) [file pone.0326339.s009.docx]

Table S9. Meta-Regression, AI^[[1]](#footnote-1)^ on Internal Validation Medial Meniscus

| Parameter | Category | Number of studies in each category | Sensitivity[95%CI] | P-value | Specificity[95%CI] | P-value |
| --- | --- | --- | --- | --- | --- | --- |
| View | Yes | 6 | 0.89 [0.85 - 0.93] | 0.02 | 0.90 [0.84 - 0.97] | 0.83 |
|  | No | 18 | 0.78 [0.71 - 0.85] |  | 0.76 [0.67 - 0.86] |  |
| Data Augmentation | Yes | 2 | 0.89 [0.78 - 1.00] | 0.65 | 0.98 [0.94 - 1.00] | 0.05 |
|  | No | 22 | 0.82 [0.76 - 0.88] |  | 0.78 [0.70 - 0.85] |  |

1. Artificial intelligence (AI) [↑](#footnote-ref-1)
