# Supplementary material for: Diagnosis of knee meniscal injuries using artificial intelligence: A systematic review and meta-analysis of diagnostic performance
Source: PLoS One. 2025 Jun 24;20(6):e0326339. doi: 10.1371/journal.pone.0326339 (PMC12186967; doi:10.1371/journal.pone.0326339)
Supplement: S10 Table — (DOCX) [file pone.0326339.s010.docx]

Table S10. Meta-Regression, AI^[[1]](#footnote-1)^ on Clinicians Internal Validation

| Parameter | Category | Number of studies in each category | Sensitivity[95%CI] | P-value | Specificity[95%CI] | P-value |
| --- | --- | --- | --- | --- | --- | --- |
| View | Yes | 16 | 0.85 [0.77 - 0.92] | 0.74 | 0.89 [0.84 - 0.93] | 0.49 |
|  | No | 1 | 0.89 [0.70 - 1.00] |  | 0.76 [0.47 - 1.00] |  |

1. Artificial intelligence (AI) [↑](#footnote-ref-1)
