# Supplementary material for: Diagnosis of knee meniscal injuries using artificial intelligence: A systematic review and meta-analysis of diagnostic performance
Source: PLoS One. 2025 Jun 24;20(6):e0326339. doi: 10.1371/journal.pone.0326339 (PMC12186967; doi:10.1371/journal.pone.0326339)
Supplement: S11 Table — (DOCX) [file pone.0326339.s011.docx]

Table S11. Meta-Regression, AI^[[1]](#footnote-1)^ on External Validation

| Parameter | Category | Number of studies in each category | Sensitivity[95%CI] | P-value | Specificity[95%CI] | P-value |
| --- | --- | --- | --- | --- | --- | --- |
| Tear | Yes | 4 | 0.82 [0.74 - 0.90] | 0.24 | 0.87 [0.84 - 0.90] | 0.00 |
|  | No | 1 | 0.82 [0.68 - 0.97] |  | 0.90 [0.88 - 0.92] |  |
| View | Yes | 3 | 0.80 [0.71 - 0.90] | 0.21 | 0.85 [0.81 - 0.89] | 0.00 |
|  | No | 1 | 0.91 [0.77 - 1.00] |  | 0.96 [0.91 - 1.00] |  |

1. Artificial intelligence (AI) [↑](#footnote-ref-1)
