# Supplementary material for: Diagnosis of knee meniscal injuries using artificial intelligence: A systematic review and meta-analysis of diagnostic performance
Source: PLoS One. 2025 Jun 24;20(6):e0326339. doi: 10.1371/journal.pone.0326339 (PMC12186967; doi:10.1371/journal.pone.0326339)
Supplement: S12 Table — (DOCX) [file pone.0326339.s012.docx]

| **Parameter** | Coefficient | CI^[[1]](#footnote-1)^ | P-value |
| --- | --- | --- | --- |
| **Algorithms Internal Validation, All studies** | 0.13 | (-6.18 – 6.45) | 0.001 |
| Medial meniscus | -6.72 | (-11.12 – -2.32) | 0.004 |
| Lateral Meniscus | -8.24 | (-13.61 – -2.86) | 0.004 |
| **Algorithms External Validation, All studies** | 8.39 | (-63.24–80.04) | 0.734 |
| **Clinicians Internal Validation, All studies** | -14.83 | (-24.81 – -4.85) | 0.006 |
| Medial meniscus | -9.87 | (-16.66 – -3.08) | 0.016 |
| Lateral Meniscus | -13.76 | (-34.88 – 7.35) | 0.145 |

**Table S12.** Publication Bias

1. Confidence interval (CI) [↑](#footnote-ref-1)
